# Protocol for genomic surveillance of *Plasmodium falciparum* antigens using amplicon-based PacBio long-read sequencing

**DOI:** 10.1016/j.xpro.2025.104093

**Published:** 2025-09-15

**Authors:** David Fernando Plaza

**Affiliations:** 1 Division of Infectious Diseases, Department of Medicine Solna, Karolinska Institutet, 17177 Stockholm, Sweden; 2 Department of Infectious Diseases and Center for Molecular Medicine, Karolinska University Hospital, 17176 Stockholm, Sweden

**Keywords:** Bioinformatics, Genomics, Sequencing, Microbiology, Molecular Biology

## Abstract

Here, we present a protocol that identifies and classifies structurally diverse variants of *msp1*, *msp2*, *glurp*, and *csp* in *Plasmodium falciparum* using an amplicon-based long-read sequencing platform. We describe steps for PCR barcoding, PacBio circular consensus sequencing (CCS), *in silico* PCR-based size variant calling, and advanced data analysis in Galaxy. By resolving full-length sequences for each antigenic clone, this approach measures infection complexity, constructs isolate phylogenies, and supports vaccine design.

For complete details on the use and execution of this protocol, please refer to Plaza et al.^1^

## Highlights

• Steps for PCR barcoding of *msp1*, *msp2*, *glurp*, and *csp* amplicons
• Instructions for SMRTbell library prep and PacBio HiFi sequencing of pools
• Guidance on Galaxy workflows for size and sequence variant calling
• Steps for phylogenetic analysis and MOI estimation from full-length reads

## Abstract (editor highlights)

Publisher’s note: Undertaking any experimental protocol requires adherence to local institutional guidelines for laboratory safety and ethics.

## Graphical abstract

**Figure:**
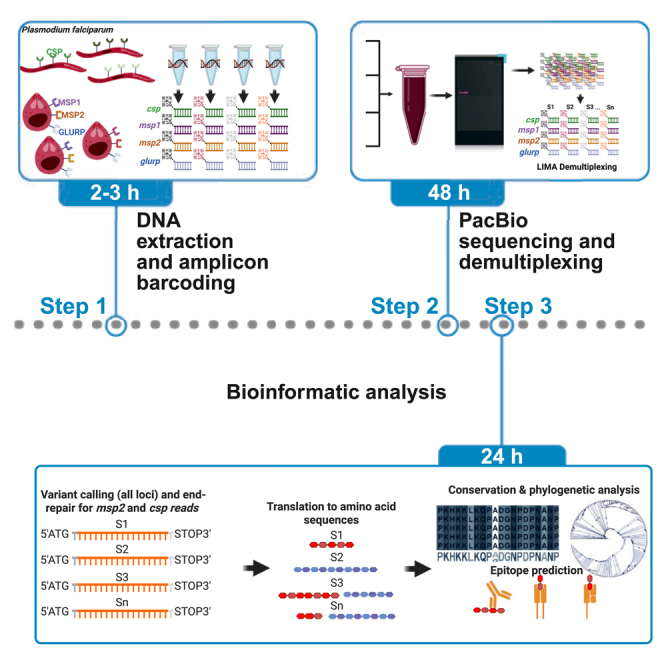

## Before you begin

This protocol describes an amplicon-based long-read sequencing strategy for *Plasmodium falciparum* antigens (merozoite surface protein 1 (*msp1*), merozoite surface protein 2 (*msp2*), glutamate rich protein (*glurp*), and circumsporozoite protein (*csp*)). It combines polymerase chain reaction (PCR) barcoding with circular consensus sequencing (CCS) to resolve antigenic diversity at single-nucleotide resolution.

### Innovation

The protocol introduces a full-length barcoding strategy that converts four polymorphic *Plasmodium falciparum* antigens (*msp1*, *msp2*, *glurp*, *csp*) into HiFi-grade amplicons ready for PacBio multiplex sequencing in ≤72 h. By coupling 40 asymmetric barcodes to high-fidelity primers, we tag up to 384 clinical isolates, eliminating the need for nested amplifications, cloning, and sequencing of individual clones in separate reactions. The resulting circular-consensus (CCS) reads capture each clone’s entire open reading frame, so size-based alleles and single-nucleotide variants are resolved simultaneously, something capillary electrophoresis or short-read panels cannot deliver. We also provide an open-source, two-stage Galaxy pipeline that (i) learns background noise directly from water controls and (ii) automatically produces size-variant matrices, clone-resolved phylogenies, T-cell epitope maps, and multiplicity-of-infection (MOI) estimates. All intermediate files are human-readable, enabling transparent troubleshooting and rapid reuse in other laboratories without command-line expertise. Finally, because barcode collections and analysis scripts are fully modular, the method can be retargeted to additional blood-stage or vector-stage antigens, or to any pathogen with indel-rich surface genes, by redesigning the primer collection and doing minor adjustments to the analysis pipelines. Together, these advances provide a scalable platform for genomic surveillance and rational vaccine design in malaria and beyond.

Before starting, ensure that the following preparatory steps, permissions, and materials are in place.

1. Confirm Institutional Permissions.
  a. Apply for biosafety approval for the work with *Plasmodium falciparum*, a microorganism that can cause infection in humans, from the relevant authorities in your country (e.g. The Swedish Work Environment Authority).
  b. Obtain approval from the relevant ethical committees for using human blood or parasite samples.
  c. Secure informed consent documentation and adhere to institutional regulations if working with clinical isolates or human-derived materials.
2. Barcoding Scheme, Primer Design and Validation.
  a. Obtain or synthesize the necessary barcoding primer sets. One primer pair amplifies and barcodes the full-length gene (for *msp2*, *msp1*, *glurp*, or *csp*).
  b. Assign barcodes to individual clinical isolates or controls, planning a unique forward and reverse barcode combination per sample to allow posterior sample demultiplexing (up to 384-plex if needed). Use the primer sets from Plaza *et al.*,^1^ these primers can be found in Table S1**.**
  c. If designing primers targeting loci different than *msp2*, *msp1*, *glurp*, or *csp*, verify *in silico* that annealing sites align with conserved regions of the antigen to avoid dropout from sequence polymorphisms.
  d. Keep track of each barcode pairing in a manifest or spreadsheet to prevent mix-ups during library prep.

**CRITICAL:** For primer sets targeting genes not included in this protocol (e.g. other surface antigens or drug resistance genes), test efficiency on control samples (e.g., synthetic sequences, cultured parasite or isolates with known reference alleles) to confirm adequate PCR yield and specificity.

3. DNA Extraction and Initial QC.
  a. Extract parasite DNA from frozen, clinical isolate-derived, packed blood cells or other sample sources (e.g., dried blood spots) using a commercial extraction kit of your preference (e.g., QIAamp DNA blood mini kit Cat. No./ID: 51106). For low-parasitemia samples, nested PCR could improve the detection of rare templates.
  b. Measure DNA integrity and concentration (e.g.,NanoDrop, Qubit). Store DNA at −80°C until ready for amplification.

**CRITICAL:** Residual contaminants (e.g., hemoglobin) can inhibit PCR. Consider extracting blood into heparin tubes. Ethylenediaminetetraacetic acid (EDTA), a common anticoagulant, can chelate and make unavailable the Mg required by enzymes used in downstream preparations (e.g. polymerase in PCR reactions). If using challenging sample types, adopt protocols (e.g., QIAamp kits) that maximize purity.

4. Prepare a Mock-Infection Control Set (Optional).
  a. If evaluating sensitivity or specificity, construct “mock infections” by mixing synthetic gene alleles in varying ratios and concentrations. This method has been standardized with concentrations ranging from 1 to 1 × 10^5^ copies/μL of synthetic *msp2* alleles.
  b. Assign dedicated barcode combinations to these controls and run them in parallel during library prep and sequencing to set variant-calling thresholds and false discovery rates.
5. Plan Data Analysis Environment.
  a. Set up and sign up for local or cloud-based bioinformatics tools (LIMA, Galaxy, etc.) for demultiplexing, size-variant calling, and sequence analysis.
  b. Confirm that you have the required packages installed in your Galaxy instance (e.g., BLAST+, MAFFT, IQ-TREE) and enough computing resources for batch processing of large CCS datasets.

**CRITICAL:** Consistent tool versions and stable compute resources help avoid pipeline errors or inconsistent variant calls.

6. Gather Additional Reagents and Equipment.
  a. Confirm availability of high-fidelity polymerases, PCR consumables, PacBio library prep kits, and a PacBio Sequel (or Revio) sequencing platform (or an equivalent sequencing provider).
  b. Secure access to a thermocycler capable of multi-well (96 or 384 wells) PCR, and a dedicated PCR workspace to reduce contamination risks.

After confirming these prerequisites, you are ready to initiate the amplicon barcoding, and CCS workflow detailed in the steps below.

### Institutional permissions

All procedures involving human blood samples in the publication presenting data supporting this protocol (Plaza *et al.*^1^) were approved by the Regional Ethical Committee in Stockholm, Sweden (approval number 00–084, 2016/1688-32) and the Ethical Review Board of the National Institute for Medical Research in Tanzania (approval number NIMR/HQ/R.8a/Vol. lxl 957). Informed consent was obtained from all study participants and/or their guardians, as appropriate. Individuals intending to replicate or adapt any part of this protocol are advised to seek approval from their relevant institutional review boards or ethics committees, in accordance with local and national regulations.

### Primer procurement and setup

**Timing: 1–2 h**

For this protocol, barcoded primers have already been designed to amplify and tag *msp1*, *msp2*, *glurp*, or *csp* in a single PCR step using Serial Cloner (open access, version 2.6). You can find the complete oligo list in the supplementary materials.

7. Obtain the barcoding primers.
  a. Retrieve the recommended forward and reverse barcoded primers for each target gene from the Table S1**.**
  b. Confirm that each primer pair is unique and specific to its corresponding sample or well. You are encouraged to use the barcode combinations shown in Figure 1. Nevertheless, other combinations are possible.

**Figure 1:**
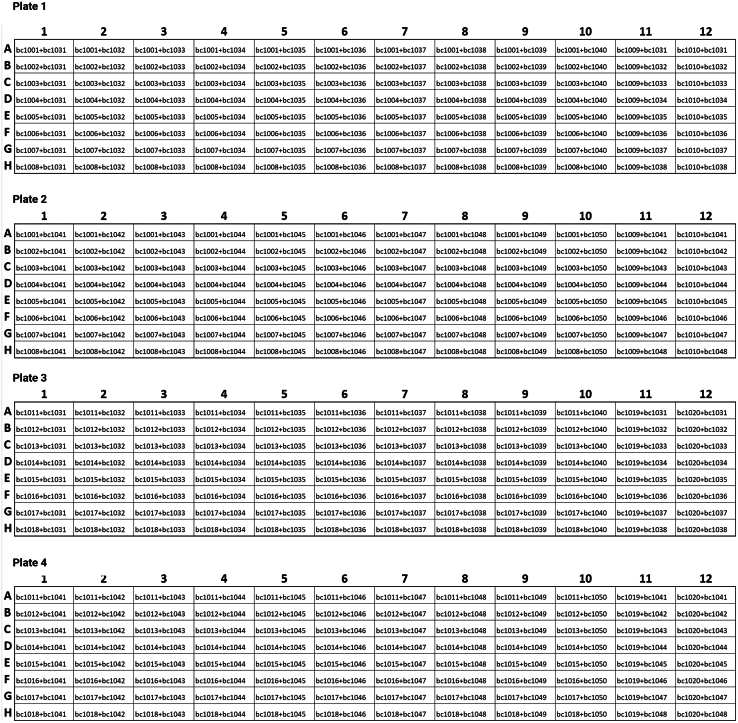
Barcoding scheme Scheme shows unique forward and reverse barcode combinations.
  c. Order the oligos as high-performance liquid chromatography (HPLC)-purified products from a reputable commercial supplier.
8. Prepare and store primer stocks.
  a. Resuspend lyophilized primers to 100 μM in nuclease-free water or Tris-EDTA (TE) buffer.
  b. Prepare a working dilution (e.g., 10 μM) for routine PCR setup; maintain these aliquots at −20°C.

**CRITICAL:** Keep a thorough list or plate map linking each primer set (barcode combination) to its designated sample or well to avoid mislabeling.

9. (Optional) Preliminary test on reference DNA.
  a. If time and material permit, amplify a known laboratory strain (e.g., 3D7, HB3) using the barcoded primers.
  b. Verify that a distinct band appears at the expected size.
  c. Proceed with barcoding of your clinical or field samples once specificity is confirmed.

If samples fail to amplify or show minimal yield, see the troubleshooting section for suggestions, including a two-step nested approach under low-DNA conditions.

### DNA extraction and QC

**Timing: 1–3 h (depending on sample throughput)**

10. Process Packed Red Blood Cells or Filter Paper.
  a. If using frozen blood pellets, thaw them on ice and transfer each pellet into a 1.5 mL microcentrifuge tube.
  b. For filter paper samples, cut out a small circle (∼3–5 mm) using a sterile punch or scissors. Place each piece into its own microcentrifuge tube.

**CRITICAL:** Avoid cross-contamination by using new gloves, fresh blades or punches, and clean surfaces between samples.

11. Perform Column-Based DNA Extraction.
  a. Use a commercial kit (e.g., QIAamp DNA Blood Mini Kit) following the manufacturer’s instructions (Figure 2).
    i. Briefly, lyse cells or filter paper discs in the provided buffer with Proteinase K.
    ii. Incubate at 56°C (∼10–15 min) to ensure complete lysis.

**Figure 2:**
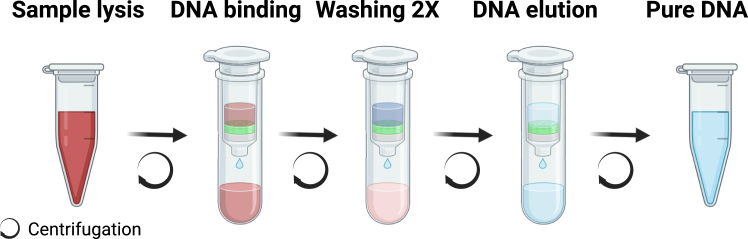
Standard spin-based DNA extraction Details may vary depending on the kit manufacturer.
  b. Pass the lysate through a spin column by centrifugation at 6000 × g (or as specified by the manufacturer of the kit).
    **CRITICAL:** Avoid touching the inner walls of the column when adding lysate to reduce carryover contamination.
    i. Discard the flowthrough and repeat wash steps as instructed.
  c. Elute DNA in 50–100 μL of the kit’s elution buffer or nuclease-free water.
    **CRITICAL:** For maximum yield, pre-warm the elution buffer to 56°C and allow it to sit on the column for 1–2 min before centrifugation.
12. Measure DNA Concentration and Purity.
  a. Use a spectrophotometer or fluorometer (e.g., Nanodrop or Qubit) to quantify extracted DNA. A 200 μL sample of whole blood from a healthy individual will typically yield 3–12 μg of DNA.
  b. Check the A260/280 ratio (for Nanodrop) to confirm a ratio of ∼1.8, indicating adequate purity.
    i. Ratios <1.6 may suggest protein or chemical contamination; consider re-purifying in that case.

**CRITICAL:** Store extracted DNA at −20°C or −80°C if you will not proceed immediately to barcoding PCR. Keep repeated freeze-thaw cycles to a minimum.

13. Record Sample Metadata (Optional).
  a. For clinical isolates, log sample IDs, extraction dates, and Qubit/Nanodrop readings in a shared spreadsheet.
  b. Link each DNA stock to subsequent barcode primer sets used in the amplification step.

### Mock infections or controls (optional)

**Timing: 1–3 h**

14. Order Synthetic Construct Controls.
  a. Identify target antigens (e.g., *msp2*) and retrieve reference sequences from PlasmoDB (https://plasmodb.org/plasmo/app) or another database.
  b. Synthesize plasmid constructs (e.g., pUC57) encoding different allelic variants to mimic multi-strain infections.

**CRITICAL:** Verify each construct’s sequence fidelity to minimize confounding by undesired mutations by Sanger sequencing using plasmid-specific primers. The manufacturer often provides quality control documentation.

**Figure 3:**
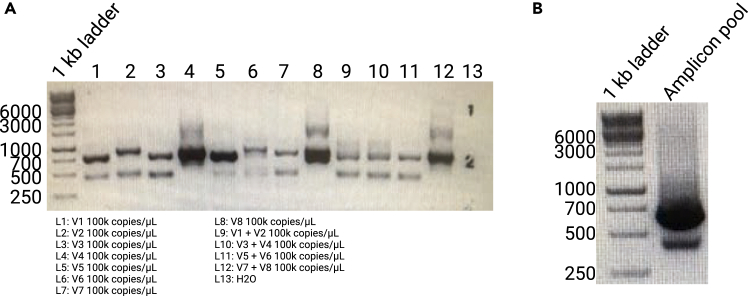
Example agarose gels used for quality control during library construction (A) Example agarose gel showing the amplification of a selection of mock infections with synthetic DNA for different variants of *msp2*. (B) Barcoded amplicon pool for *msp2* and used for SMRTbell library construction.
  b. Document the allele composition, concentration, and barcode assignments for each mixture in a dedicated spreadsheet.

15. Prepare Mixtures of Mock Infections.
  a. Dilute each plasmid (or linearized DNA) to the desired template concentration (e.g., 1–10000 copies/μL).
    i. Use sterile water or TE buffer to maintain DNA stability.
  b. Combine 2–10 allelic variants in one tube in concentrations ranging from 0.002 fM (approximately 1 copy/μL) to 20 fM (approximately 10000 copy/μL), to emulate mixed *P. falciparum* infections of varying complexity.
    **CRITICAL:** Record the molar ratio of each variant to track specificity or sensitivity thresholds during analysis.
    i. For instance, if testing the lower limits of detection, add one variant at 1 copy/μL in the presence of others at higher copy numbers.
  c. Aliquot each mock-infection mixture into 1.5 mL microcentrifuge tubes and store at −20°C or −80°C until use.
16. QC and Documentation.
  a. (Optional) Run an initial PCR on each mixture to confirm that the expected variants are amplified (Figure 3).

**CRITICAL:** Keep these records consistent with subsequent library prep steps to match observed sequences with known input ratios.

***Note:*** These synthetic controls enable you to establish variant-calling thresholds, estimate false-positive rates, and confirm pipeline performance before analyzing clinical *P. falciparum* isolates.

## Key resources table

**Table:** 

REAGENT or RESOURCE	SOURCE	IDENTIFIER
**Biological samples**		

Venous blood from volunteer study subjects	Farnert et al.^2^	N/A

**Chemicals, peptides, and recombinant proteins**		

Phusion Hot Start II DNA polymerase	Thermo Scientific	F549L
SOC Broth	Millipore	85469
LB Broth with agar (Lennox)	Merck	L2897
GelRed® Nucleic Acid Stain 10000X DMSO	Millipore	SCT122
Deoxynucleotide triphosphate (dNTP) mix (10 mM each) 1 mL	Thermo Scientific	R0192

**Critical commercial assays**		

QIAamp DNA blood mini kit	QIAGEN	Cat. No. / ID: 51106
QIAquick PCR purification kit	QIAGEN	Cat. No. / ID: 28106
QIAquick Gel Extraction Kit	QIAGEN	28704
CloneJET™ PCR Cloning Kit with DH10B Competent Cells, 20 Reactions	Thermo Scientific	K123120
Qubit 1× dsDNA HS Kit	Thermo Scientific	Q33231
1 Kb Plus DNA Ladde	Invitrogen	10787026
SMRTbell template prep kit 3.0	Pacific BioSciences	100-259-100

**Deposited data**		

Raw FASTQ files	This paper	ENA study: PRJEB46950, samples ERS12142703 - ERS12143079, ERS15546120 - ERS15546494 and ERS15547516 - ERS15547539

**Oligonucleotides**		

msp2_fw: 5′-ATGAAGGTAATTAAAACATTGTCTATTATA-3′	Snounou et al.^3^	N/A
msp2_rv2: 5′-TTATATGAATATGGCAAAAGATAAAACAA-3′	This paper	N/A
Oligonucleotides for amplicon barcoding of *msp1*, *msp2*, *glurp*, and *csp* (see Table S1)	This paper	N/A

**Recombinant DNA**		

pUC57-msp2_HB3	This paper	KEGG: PFHG_00788
pUC57-msp2_CD01	This paper	PlasmoDB: PfCD01_020011700
pUC57-msp2_ Dd2	This paper	PlasmoDB: PfDd2_020009600
pUC57-msp2_SN01	This paper	PlasmoDB: PfSN01_020009800
pUC57-msp2_KE01	This paper	PlasmoDB: PfKE01_020009300
pUC57-msp2_SD01	This paper	PlasmoDB: PfSD01_020012300
pUC57-msp2_GN01	This paper	PlasmoDB: PfGN01_020012100
pUC57-msp2_7G8	This paper	PlasmoDB: Pf7G8_020011500
pUC57-msp2_GB4	This paper	PlasmoDB: PfGB4_020009800
pUC57-msp2_3D7	This paper	PlasmoDB: PF3D7_0206800

**Software and algorithms**		

Galaxy Europe	Afgan et al.^4^	https://usegalaxy.eu/
Analysis pipeline for Galaxy (workflow 1 and workflow 2 at different false-positive rates) and accessory files	This paper	https://doi.org/10.5281/zenodo.15762538
SMRT Link toolbox (version 10.0.0.108728)	Pacific BioSciences	https://www.pacb.com/support/software-downloads/
Serial Cloner (v2.6)	SerialBasics	http://serialbasics.free.fr/Serial_Cloner.html
BLAST+	Camacho et al.^5^; Cock et al.^6^	https://ftp.ncbi.nlm.nih.gov/blast/executables/blast+/LATEST/
MAFFT Galaxy version 7.489+galaxy0	Katoh et al.^7^	https://mafft.cbrc.jp/alignment/software/
IQ-TREE Galaxy version 2.1.2+galaxy2	Nguyen et al.^8^	http://www.iqtree.org
IEDB MHC Galaxy version 2.15.2	Dhanda et al.^9^	http://tools.iedb.org/mhcii/download/

**Other**		

Thermocycler	Different manufacturers	N/A
PacBio Sequel II/Revio	PacBio	N/A
Magnetic rack	Different manufacturers	N/A
Bioanalyzer/TapeStation	Agilent	G2992AA
UV or blue-light transilluminator	Different manufacturers	N/A
Qubit fluorometer	ThermoFisher Scientific	N/A

## Materials and equipment

The following optional items and setup details may facilitate efficient protocol execution and ensure consistent results.

• PCR Reaction Master Mixes.
  ○ Prepare master mixes fresh to minimize polymerase degradation.
  ○ Aliquot immediately before use and keep on ice during pipetting.
  ○ Avoid multiple freeze-thaw cycles for any enzyme solution.
• Barcoding Primer Storage.
  ○ Maintain 10 μM working dilutions in microcentrifuge tubes at −20°C.
  ○ Prepare fresh aliquots for library construction to avoid repeated freeze-thaw.
  ○ In four 96-well plates per locus (e.g. 4 plates for *msp1*, 4 plates for *msp2*, etc.) and following the provided barcoding plate map, mix equal amounts of the 10 μM stocks of forward and reverse barcoding primers in each well to a final volume of 50 μL (25 μl of each 10 μM barcoding primer).
    - The downstream setup of barcoding reactions requires 5 μL of these pre-plated barcoding primer mixes; therefore, a 50 μl premix provides enough primer for approximately 10 libraries (each library of 384 samples and controls).
    - Thoroughly label each plate to keep track of the barcodes included (Figure 1).
• Custom Software/Computing Environment.
  ○ Run demultiplexing and barcode removal scripts for LIMA in Conda.
  ○ Use a locally installed or cloud-based Galaxy instance to run the provided analysis pipelines.
    - Back up data and parameter settings regularly to ensure data integrity and reproducibility.
• Alternative Materials.
  ○ If Phusion Hot Start II is unavailable, other high-fidelity polymerases may be used. Nevertheless, PCR conditions might require adjustments according to the manufacturer’s instructions.
• Storage Conditions.
  ○ Keep prepared master mixes on ice for immediate use; do not store them long-term once enzymes or dNTPs are added.
  ○ Return polymerase stocks to −20°C promptly after pipetting to preserve enzyme activity.
  ○ Dispose of all biological or chemical waste in accordance with institutional biosafety regulations.

## Step-by-step method details

### Whole-gene (full-length) barcoding PCR

**Timing: 2–3 h per locus**

This step amplifies ∼700–1,500 bp regions of the target loci (e.g., *msp1*, *msp2*, *glurp*, or *csp*), corresponding to complete open reading frames for *msp2* and *csp* and large gene fragments for *msp1* and *glurp* in a single 50 μL reaction, using barcoded forward and reverse primers for downstream demultiplexing.

1. Prepare the PCR master mix.
  a. Thaw all reagents on ice, including 5× HF buffer, dNTPs, plates with barcoded primer mixes, and polymerase.
  b. Vortex briefly and spin down to collect.
  c. Combine reagents for 50 μL reactions according to the master mix table in a 50 mL centrifuge tube (see materials and equipment setup).
  d. Keep the master mix on ice until loading into PCR plates or tubes.

**Table:** 

Reagent	Final concentration	Volume/Reaction	Storage/Notes
5× Phusion HF Buffer	1×	10.0 μL	Store at 4°C; stable up to 6 months (per manufacturer)
10 mM dNTP mix	200 μM (each)	1.0 μL	Aliquot, store at −20°C
Phusion Hot Start II (2 U/μL)	0.02 U/μL	0.5 μL	Keep at −20°C; minimize time at RT
Nuclease-free water	–	32.5 μL	Molecular-biology grade
Barcoding Primer Mix (Fw & Rv, each 10 μM)	0.5 μM (each)	5.0 μL	Combine forward & reverse barcodes at 1:1; store at −20°C
**Purified genomic DNA**	–	1.0 μL (5–50 ng)	Nested template; store at −20°C

Total reaction volume: 50 μL. Preparation: Keep reagents on ice, mix thoroughly, and use immediately.

2. Dispense master mix and add template DNA using appropriate multichannel pipettes.
  a. Aliquot 44 μL master mix into each well.
  b. Add 5 μL barcoding primer mix (to a final 0.5 μM for each oligo) to each well.
  c. Add 1 μL genomic DNA template (∼1–20 ng) to each reaction last.

**CRITICAL:** Seal the plate or close tubes carefully to prevent cross-contamination; mix gently by tapping or brief centrifugation.

3. Run the thermocycler program.
  a. Use the following settings (or adapt based on polymerase recommendations):
    **Table:** Amplification conditions for barcoding

Steps	Temperature	Time	Cycles
Initial Denaturation	98°C	30 s	1
Denaturation	98°C	10 s	5 cycles
Annealing	50°C	30 s	
Extension	72°C	40 s	
Denaturation	98°C	10 s	35 cycles
Annealing and Extension	72°C	40 s	
Final extension	72°C	5 min	1
Hold	4°C	forever
  b. (Optional) If samples are known to be low parasitemia or difficult to amplify, see troubleshooting for details on adding a preliminary 15 μL reaction.
  c. After cycling is complete, proceed immediately to Amplicon Verification on Agarose Gels or store plates with reactions at 4°C if short-term, −20°C if long-term.

### Amplicon verification on agarose gels

**Timing: 30–45 min**

This step confirms that barcoded PCR products are present at the expected size range (typically ∼700–1,500 bp, depending on the antigen) and ensures that yield is adequate for downstream library preparation.

4. Prepare the agarose gel.
  a. Weigh out 1.0–1.2 g agarose (1%–1.2% w/v), add to an Erlenmeyer flask, and add 100 mL 1× Tris-borate-EDTA (TBE) or 1× Tris-acetate-EDTA (TAE) buffer.
  b. Microwave or heat until the agarose is fully dissolved, swirling gently and regularly to prevent boil-over.
  c. Cool the molten agarose to ∼50°C, then add a suitable DNA stain (e.g., GelRed) according to the manufacturer’s instructions.
  d. Pour the solution into a gel tray fitted with a comb; allow 20–30 min for the gel to solidify at 20°C–24°C.
5. Load and run the gel.
  a. Place the solidified gel in the electrophoresis chamber and fill with 1× running buffer so the gel is fully submerged.
  b. Mix 3–5 μL PCR product with 1 μL 6× loading dye, and load the entire volume (∼4–6 μL) into each lane.
  c. Include a DNA ladder (e.g., 1 kb Plus) in at least one lane for sizing.
  d. Run the gel at 100–120 V for ∼30 min, or until the dye front migrates 2/3 of the gel length.
6. Visualize and document band sizes.
  a. Carefully remove the gel and view under a ultraviolet (UV) or blue-light transilluminator (depending on the stain used).
  b. Confirm that distinct bands of the expected amplicon size are present; faint secondary bands may indicate nonspecific amplification.
  c. (Optional) Take a photograph or digital image for record-keeping (see example in Figure 3).
  d. If yields are too low, consult troubleshooting or consider an initial 15 μL nested PCR.

### Optional pJET cloning and colony PCR (quality control)

**Timing: 2–3 days**

This optional step verifies amplicon accuracy by cloning PCR products into a plasmid vector and sequencing individual clones. This approach helps detect potential chimeras, artifactual variants or unilateral amplicon barcoding.

7. Prepare the pJET ligation reaction.
  a. Purify the amplicon band of interest (∼700–1,500 bp) from agarose using a commercial gel extraction kit (e.g., QIAquick Gel Extraction Kit).
  b. Use a blunt-end cloning kit (e.g., Thermo Scientific CloneJET PCR Cloning Kit) following the manufacturer’s instructions.
  c. Combine ∼50–100 ng purified PCR product with 1× ligation buffer and 1 μL pJET1.2/blunt cloning vector in a final volume of 10 μL.
  d. Incubate the ligation mixture for 5 min at 20°C–24°C (or as recommended by the kit).
8. Transform competent cells and plate.
  a. Thaw chemically competent E. coli (e.g., DH10B) on ice for 15 min.
  b. Add 5 μL of the ligation reaction to 50 μL competent cells; swirl gently.
  c. Incubate on ice for 20–30 min, then heat-shock at 42°C for 45 s.
  d. Immediately return tubes to ice for 2 min, then add 250 μL Super Optimal with Catabolite repression (SOC).
  e. Let the cells recover under shaking at 37°C for 1 h (200–250 rpm), and plate 100–200 μL on LB-agar plates containing the appropriate antibiotic.
  f. Grow for 18 h at 37°C.
9. Pick colonies and perform colony PCR.
  a. Select 5–10 colonies per plate to sample amplicon diversity.
  b. Streak each colony onto a fresh antibiotic plate for stock reference.
  c. Resuspend a small amount of each colony in 20 μL nuclease-free water.
  d. Set up a 15 μL or 25 μL colony PCR using vector-specific primers (e.g., pJET forward and reverse) according to the manufacturer’s protocol.
  e. Run thermocycler: 30 cycles of 95°C (30 s), 55°C (30 s), and 72°C (1 min per kb).
  f. Verify insert size on a 1% agarose gel.
  g. (Optional) Purify positive colony PCR products and submit for Sanger sequencing to confirm allele identity.

### Amplicon pooling and cleanup

**Timing: 1–2 h**

This step combines barcoded PCR products from multiple wells (e.g. all the wells in a plate row) into a consolidated pool, then removes excess primers, dNTPs, and other contaminants by a standard column-based (or bead-based) cleanup (e.g. eight clean-up preps for a 96-well plate). Thereafter, eluted products are pooled further and are then suitable for library preparation.

10. Pool barcoded PCR products.
  a. Collect the total 50 μL reaction volume from each sample well in an individual plate row (12 wells or 600 μL total) into a labeled 1.5 mL or 2.0 mL microcentrifuge tube.
11. Perform column-based cleanup.
  a. Use a commercial PCR purification kit (e.g., QIAquick PCR Purification Kit) following the manufacturer’s protocol.
  b. Adjust volumes as necessary to avoid exceeding column capacity (e.g., split pools over multiple loads).
  c. Elute pooled amplicons in 30–50 μL of elution buffer (EB) or nuclease-free water.
  d. Pool the resulting eluted samples into a single locus-specific pool (e.g. 32 elution tubes yielding a 960 μL pool for a 30 μL elution volume). Measure concentration and A260/280 ratio.
  e. In a 1.5 mL, mix equal amounts (in ng) of the four locus-specific amplicon pools, at least 250 ng of each (1 μg in total as a minimum) or the amount required by your core facility or commercial sequencing provider.
12. Assess quality of the pooled amplicon.
  a. Store the cleaned pooled DNA at −20°C before sequencing.

### Library preparation and PacBio sequencing (SMRTbell prep kit 3.0)

**Timing: ∼3.5 h hands-on (∼6 h elapsed, not including optional safe stops)**

This step converts the pooled, cleaned amplicons into a polymerase-bound SMRTbell library and loads it on a PacBio HiFi instrument. The workflow follows PacBio’s *Preparing multiplexed amplicon libraries using SMRTbell prep kit 3.0* (PN 102-359-000) with minor volume adjustments for 384-sample pools.

13. Verify input quantity and size, then perform a 1.3 × bead cleanup.
  a. Measure DNA concentration with the Qubit 1× dsDNA HS kit (Thermo Fisher) and record total nanograms in the pool.
  b. Verify fragment size on a TapeStation or BioAnalyzer; confirm the modal length for *msp1*, *msp2*, *glurp*, *csp* pools.
  c. Transfer the required DNA mass to 0.2 mL strip tubes (≥100 ng per 1–3 kb pool for Sequel II/e).
  d. Add 1.3 × volume of 20°C–24°C SMRTbell cleanup beads, mix, and incubate 10 min at 20°C–24°C.
  e. Place tubes on a magnetic rack, wash beads twice with 80% ethanol, air-dry ≤30 s, and elute DNA in 47 μL Low TE.
  f. Safe stop: store eluate at 4°C for up to 1 week.
14. Repair and A-tail the pooled DNA.
  a. Combine the Repair Master Mix on ice (8 μL Repair buffer + 4 μL End-repair mix + 2 μL DNA-repair mix per pool).

**Table:** 

Reagent	Volume (μL)
Repair buffer	8
End repair mix	4
DNA repair mix	2
**Total volume**	14
  b. Add 14 μL mix to 46 μL DNA (total 60 μL), pipette-mix, centrifuge, and incubate the reaction in a thermocycler: 30 min at 37°C, then 5 min at 65°C and finally 4°C on hold.
15. Ligate SMRTbell adapters and perform a cleanup.
  a. Prepare Ligation Master Mix (4 μL SMRTbell adapter + 30 μL Ligation mix + 1 μL Ligation enhancer per pool).
  b. Add 35 μL mix to the 60 μL repaired sample (95 μL total), mix, quick-spin, incubate 30 min at 20°C, then hold 4°C.
  c. Bind DNA to 1.3 × cleanup beads (124 μL), wash twice with 80% ethanol, elute in 40 μL Elution buffer, and centrifuge shortly.
  d. Safe stop: store adapter-ligated library at 4°C for up to 2 weeks (−20°C for long-term storage).
16. Nuclease treatment and second cleanup.
  a. Prepare Nuclease Master Mix (5 μL Nuclease buffer + 5 μL Nuclease mix).
  b. Add 10 μL mix to 40 μL library (50 μL total), mix, centrifuge shortly, incubate 15 min at 37°C, hold 4°C.
  c. Bind DNA to 1.3 × beads (65 μL), wash twice with 80% ethanol, elute in 26 μL Elution buffer, quick-spin, and quantify 1 μL on a Qubit instrument.
  d. Normalize to ≤10 ng μL^−1^ (for 1–3 kb libraries) using Elution buffer.
17. Anneal the sequencing primer, bind polymerase, and do a post-binding cleanup (ABC).
  a. Combine 25 μL library with 12.5 μL Annealing buffer + 12.5 μL Standard sequencing primer; incubate for 15 min at 20°C–24°C.
  b. On ice, prepare Polymerase Dilution (47 μL Polymerase buffer + 3 μL Sequencing polymerase).
  c. Add 50 μL of the polymerase dilution to the annealed library (100 μL total), mix, incubate 15 min at 20°C–24°C.
  d. Add 130 μL cleanup beads, incubate during 10 min, place on magnet, discard supernatant, and elute in 25 μL light-protected Loading Buffer 96 (50 μL if using non-SPRQ kits).
  e. Determine final concentration with a Qubit instrument; aim for a 200–300 pM loading concentration (use SMRT Link v.13.3 Loading Calculator).
18. Load and run the PacBio sequencer
  a. Transfer the polymerase-bound library to the sequencing reagent plate (25 μL per SMRT Cell).
  b. Follow SMRT Link v13.3+ Sample Setup for the chosen platform (Revio SPRQ, Vega, or Sequel II/e) and launch a ≥30 h movie for HiFi mode.
  c. Safe stop: polymerase-bound libraries are stable 1 month at 4°C or 6 months at −20°C (≤4 freeze–thaw cycles).

Refer to PacBio protocol PN 102-359-000 for detailed reagent handling, thermocycler programs, and platform-specific loading guidance.

### Data analysis and variant calling

**Timing: 24 h (variable, depending on computational resources)**

This step processes raw CCS reads through two sequential Galaxy workflows to rename samples, filter read counts, and generate final variant calls, phylogenies, and associated metrics (e.g., multiplicity of infection). The pipeline is deposited in a public Zenodo repository accompanying this protocol (https://doi.org/10.5281/zenodo.15762538).

19. Prepare the input and run Workflow 1 (Renaming and Read Counting).
  a. Obtain the demultiplexed FASTQ files (post-LIMA) for each barcoded sample.
  b. Create a two-column renaming file: Column 1 lists the existing FASTQ file names, and Column 2 assigns each barcode combination to its corresponding sample identifier.
  c. Open Galaxy (or a local instance) and upload the FASTQ collection and the renaming file.
  d. Launch Workflow 1.
    i. Provide the renaming file as input.
    ii. Verify that negative controls (e.g., water controls) are correctly recognized; the workflow calculates mean and standard deviation of reads in these controls for background filtering.
  d. Wait for Workflow 1 to complete. Record any flagged samples below or near the read coverage threshold.
20. Retrieve outputs from Workflow 1.
  a. Examine the tabular output listing each sample’s read count. Identify the recommended coverage threshold (mean water control + 2 SD).
  b. Locate the “Collapsed Collection” file, which merges read data across all FASTQs. This serves as input to Workflow 2.
  c. Make note of any samples whose read counts fall below the threshold, as they will be automatically filtered out in subsequent steps.
21. Set up Workflow 2 (Genotyping, Phylogeny, and multiplicity of infection (MOI) Analysis).
  a. Upload the following additional files to Galaxy:
    i. The FASTA files with orthologous genes used as roots in the construction of phylogenies: *msp1* and *msp2* from *P. billcollinsi*, and *glurp* and *csp* from *P. praefalciparum.*
    ii. The size variant genotyping oligos in FASTA format (e.g., ‘‘genotyping_oligos_dimorphism.fasta’’) for *msp1*, *glurp* and *msp2.*
    iii. The table with the number of reads per sample resulting from Workflow 1 and titled *Reads_per_sample_prefilter* (Figure 4).

**Figure 4:**
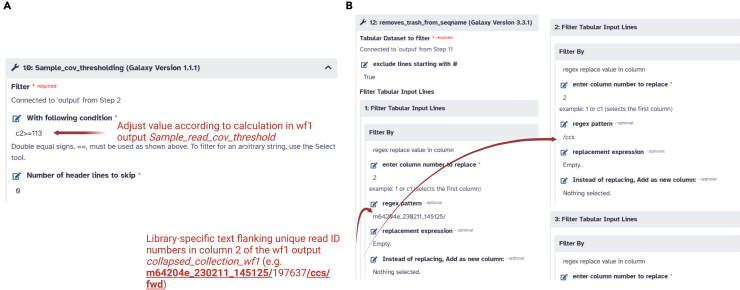
Setup of critical steps for workflow usage (A) *10: Sample_cov_thresholding* and (B) *12: removes_trash_from_seqname* of Workflow 2 in Galaxy.
  b. Launch Workflow 2.
    i. In the step 10 of workflow 2 titled *Sample_cov_thresholding*, input the coverage threshold from step 17a *With following condition* for column 2 (c2) where read counts are recorded (e.g. c2>=113).
    ii. (Optional) Adjust the number of bootstraps for phylogenetic trees if needed (default 1000).
    iii. In step 12, titled *removes_trash_from_*seqname, fill in as *filter by/regex pattern* the text flanking unique read identifiers in column 2 from the file *collapsed_collection_wf1* resulting from workflow 1 (e.g. *m64204e_230211_145125/*, */ccs*, */fwd* and */rev*) (Figure 4). These strings of text vary from library to library and, therefore, have to be input manually. Press *Run Workflow*
22. Monitor Workflow 2 progress.
  a. Galaxy displays multiple job steps (403 steps). Expect run times of several hours on average HPC resources.
  b. Proceed once all “green” (completed) steps appear in Galaxy’s history pane.
23. Collect final outputs.
  a. Download read coverage matrices for size variants, sequence variants, and filtered sample sets (e.g., *sizevar_readcov_matrix_IC_post_thresholding*).
  b. Download phylogenetic trees (e.g., IQ-TREE maximum-likelihood results) produced for the four loci genotyped.
  c. Retrieve predicted T cell epitopes (files “*Consensus_MHC_II_binding_only*” and “Consensus_MHC_II_binders_rank_equal_or_lower_to_1_percent”) and multiplicity-of-infection estimates (file “*MOI_c2sizemsp1_c3seqmsp1_c4sizemsp2_c5seqmsp2_c6sizeglurp_c7seqglurp*”).

## Expected outcomes

The expected outcome of this protocol is a high-quality amplicon sequencing dataset capturing the full-length diversity of targeted *P. falciparum* antigens (*msp1*, *msp2*, *glurp*, or *csp*). Typically, each successfully amplified sample yields a single dominant product (∼700–1,500 bp) visible on an agarose gel, although coinfected clinical isolates may contain multiple allelic variants. After library preparation and PacBio circular consensus sequencing (CCS), you can anticipate approximate 2 million high-fidelity reads per sample in a typical SequelII sequencing cell, each with a unique barcode combination. The resulting data typically allow: High-accuracy variant calls: Within-sample minor clones can be resolved down to roughly 1%–5% abundance, depending on sequencing depth and parasitemia. MOI estimates: The pipeline partitions size and sequence variants to measure how many distinct clones are present in each sample. Phylogenetic relationships: Automated maximum-likelihood analysis yields gene-level trees that group variants by similarity (e.g., IC versus FC27 families for *msp2*). Immunological insights: T cell epitope prediction highlights conserved or variable peptide stretches, potentially guiding vaccine design or epidemiological studies. Overall, the user can expect a robust, fine-grained analysis of polymorphic malaria antigens, supporting epidemiological surveillance or immunological investigation.

## Quantification and statistical analysis

This protocol’s primary data processing involves two sequential analysis workflows. The first workflow renames and counts demultiplexed reads to identify an appropriate read coverage threshold, one that estimates the amount the background noise as defined by the water (negative) controls. The second workflow applies that threshold to remove underrepresented samples, groups reads by size and sequence variants, and runs maximum-likelihood phylogenies, T cell epitope predictions, and MOI calculations.

Sample inclusion/exclusion depends on whether a sample’s read coverage exceeds the negative-control threshold (mean + 2 standard deviations of water-control reads). Any sample failing this criterion is automatically filtered out. For the retained samples, additional variant calling thresholds based on read coverage and setting up a false positive rate (FPR) of 0.001 are applied automatically as part of workflow 2. The workflow also estimates size variants (e.g., for distinct IC or FC27 families of *msp2*, or MAD20, RO33 or K1 families of *msp1*) and calls single-nucleotide variants to refine infection complexity. Phylogenetic trees are generated with 1,000 bootstrap replicates by IQ-TREE, providing branch support for each variant cluster. T cell epitope prediction (for potential immunogenic regions) uses a consensus method for the common HLA alleles DRB1∗03:01, DRB1∗07:01, DRB1∗15:01, DRB3∗01:01, DRB3∗02:02, DRB4∗01:01 and DRB5∗01:01 with a 1% percentile cutoff, while MOI is defined as the number of size and sequence variants detected above the chosen coverage threshold.

Although the pipeline supplies default FPRs of 0.001 at both the size- and sequence-variant-calling levels, researchers can modify these parameters to optimize detection for their use case. Generally, samples with robust coverage (>200 reads) yield reliable antigen diversity estimates and inform epidemiological or immunological interpretations.

## Limitations

Even though this barcoding approach yields rich data on parasite antigen diversity, several factors can limit its reliability. Extremely low-parasitemia or partially degraded DNA sometimes fails to produce an amplification product, particularly if the sample is heavily fragmented or contaminated. Additionally, genetic drift in primer-annealing regions might cause dropouts for certain alleles and hinder variant calls. The single-round PCR may introduce limited PCR artifacts such as chimeric amplicons when multiple clones coexist in the same infection, though these events are typically rare. Highly multiplexed libraries can also experience uneven coverage across samples, especially if barcode efficiencies vary. Lastly, the downstream computational workload for large-scale datasets can be high, requiring stable computing resources to complete phylogenies and variant analyses without interruption.

## Troubleshooting

### Problem 1

Low or no amplicon yield (related to steps 1–3).

When the barcoded PCR yields faint or absent bands, especially from low-parasitemia samples, you may not see distinct products on the agarose gel.

### Potential solution

• Confirm that the DNA template is intact and above 1–10 ng in each 50 μL reaction. Re-extract or concentrate samples if necessary.
• Increase the annealing temperature slightly (e.g., +2°C) or lengthen the extension time by 10–20 s.
• (Optional) Perform a pre-nesting 15 μL PCR (nested approach) if certain samples consistently fail in the direct 50 μL reaction.
• Consider redesigning or reordering specific barcoding primers displaying low yields

**Table:** Pre-nested PCR master mix for *msp2* (15 μL reactions)

Reagent	Final Conc.	Volume/Reaction	Storage/Notes
5× Phusion HF Buffer	1×	3.0 μL	Store at −20°C
10 mM dNTP mix	200 μM (each)	0.3 μL	Aliquot, store at −20°C
msp2_fw: 5′-ATGAAGGTAATTAAAACATTGTCTATTATA-3' (10 μM)	0.5 μM	0.75 μL	Store at −20°C
msp2_rv2: 5′-TTATATGAATATGGCAAAAGATAAAACAA-3’ (10 μM)	0.5 μM	0.75 μL	Store at −20°C
Phusion Hot Start II (2 U/μL)	0.02 U/μL	0.15 μL	Keep at −20°C; minimize time at RT
Nuclease-free water	–	9.05 μL	Molecular-biology grade
**Template DNA**	–	1.0 μL (5–50 ng)	Add last; store sample at −20°C

Total reaction volume: 15 μL. Preparation: Keep reagents on ice, mix thoroughly, and use immediately.

**Table:** Pre-nesting PCR program for *msp2* (15 μL reactions)

Steps	Temperature	Time	Cycles
Initial Denaturation	98°C	30 s	1
Denaturation	98°C	10 s	40 cycles
Annealing	58.1°C	30 s	
Extension	72°C	40 s	
Final extension	72°C	5 min	1
Hold	4°C	Forever	

### Problem 2

Multiple nonspecific bands or smeared products (related to steps 1–3).

Overlapping or smudged patterns on agarose gel can obscure the correct amplicon size, undermining barcoding success.

### Potential solution

• Optimize Mg2+ and primer concentrations. For some loci, 1.5–2.0 mM Mg2+ or 0.3–0.4 μM primers reduces background.
• Decrease the template concentration.
• Decrease the extension time or reduce the total PCR cycle count if smearing persists.
• Double-check that only the barcoded forward–reverse primer combination is used (avoid leftover nested primers).

### Problem 3

Incomplete pJET cloning or no positive colonies (related to steps 7–9). Sometimes no transformants grow, or resulting colonies show incorrect insert size.

### Potential solution

• Re-check blunt-end ligation protocols (e.g., ensure you used a final repair step if the polymerase leaves 3′ overhangs).
• Decrease ligation ratio if vector self-ligation is suspected (run a control with vector-only).
• Decrease the concentration of the pJET.
• Verify colony PCR conditions, and confirm the correct antibiotic was present on plates.

### Problem 4

Uneven pooling or insufficient cleanup (related to steps 10–12). If the final pooled library still contains primer dimers or shows variable concentrations, it may yield poor SMRTbell ligation and reduce sequencing coverage.

### Potential solution

• Measure each row or group’s DNA concentration separately and pool equimolarly (or at least standardize approximate amounts).
• Repeat the column purification step or use a 1:1 or 0.8:1 ratio of AMPure PB beads to remove <300 bp fragments.
• Confirm the final eluate’s purity (A260/230 > 2.0, minimal leftover ethanol).

### Problem 5

Low read counts or missing barcode labels after PacBio sequencing (related to steps 13–18).

Some samples might have insufficient CCS reads, or no reads assigned to their barcodes.

### Potential solution

• Check that each barcode pair is unique. Even a one-base mismatch between barcodes can cause misassignments.
• Increase loading concentration during SMRTbell prep.
• For extremely low-yield samples, re-run the barcoding PCR or confirm that you are not below the negative-control read threshold.

## Resource availability

### Lead contact

Further information and requests for resources and reagents should be directed to and will be fulfilled by the lead contact, David Fernando Plaza (david.plaza@ki.se).

### Technical contact

Technical questions about executing this protocol will be answered by the technical and lead contact, David Fernando Plaza (david.plaza@ki.se).

### Materials availability

• This study did not generate new unique biological reagents.
• All barcoding-primer sequences required to reproduce the protocol are provided in the Supplementary Tables and may be ordered from any commercial oligonucleotide supplier.
• Synthetic *msp2* plasmid constructs listed in the key resources table are available from the authors upon reasonable request and completion of a material transfer agreement (MTA) with Karolinska Institutet.

### Data and code availability

• Demultiplexed HiFi (CCS) FASTQ files generated with this protocol have been deposited in the European Nucleotide Archive (ENA) under study accession PRJEB46950 and are publicly accessible.
• Galaxy workflows together with accessory input files are archived on Zenodo (https://doi.org/10.5281/zenodo.15762538) and released under an open-source license.
• The published article^1^ includes all sequencing datasets, processed matrices, and code necessary to reproduce the analyses; no additional data were generated.

## Acknowledgments

I am grateful to the volunteers and field teams who made the Tanzanian isolate collection possible. This work was supported by grants from 10.13039/501100006350Stiftelsen Clas Groschinskys Minnesfond (M2334), 10.13039/100010823Tore Nilsons Stiftelse for Medicinsk Forskning (2023-131), 10.13039/501100004047Karolinska Institutet (2020-02084), and 10.13039/501100004359Vetenskapsrådet (2021-04072 and 2021-03105). Sequencing and compute were carried out with invaluable help from the National Genomics Infrastructure (NGI)/Uppsala Genome Center and UPPMAX; those facilities are financed by RFI/VR, SciLifeLab, and the Swedish National Infrastructure for Computing (SNIC, VR 2018-05973). Galaxy analyses were ran in part on infrastructure funded by CRC-992 “Medical Epigenetics” (10.13039/501100001659DFG SFB 992/1 2012) and the 10.13039/501100002347German BMBF (grants 031A538A/A538C RBC, 031L0101B/031L0101C de.NBI-epi, and 031L0106 de.STAIR).

## Author contributions

D.F.P.: conceptualization, methodology, software, validation, formal analysis, investigation, resources, data curation, writing – original draft, writing – review and editing, visualization, supervision, project administration, and funding acquisition.

## Declaration of interests

The author declares no competing interests.

## Declaration of generative AI and AI-assisted technologies in the writing process

During the preparation of this work, the author used ChatGPT in order to improve language and readability. After using this tool/service, the author reviewed and edited the content as needed and takes full responsibility for the content of the publication.

## Supplemental information

Table S1. Barcoding oligo collections for the amplification and sequencing of *Plasmodium falciparum* loci *msp1*, *msp2*, *glurp*, and *csp* related to the section Before you begin Use always HPLC-purified barcoding oligos when constructing sequencing libraries.
